# Impact of the COVID-19 Pandemic on Physical and Mental Health in Lower and Upper Middle-Income Asian Countries: A Comparison Between the Philippines and China

**DOI:** 10.3389/fpsyt.2020.568929

**Published:** 2021-02-09

**Authors:** Michael Tee, Cuiyan Wang, Cherica Tee, Riyu Pan, Patrick W. Reyes, Xiaoyang Wan, Joseph Anlacan, Yilin Tan, Linkang Xu, Chloe Harijanto, Vipat Kuruchittham, Cyrus Ho, Roger Ho

**Affiliations:** ^1^College of Medicine, University of the Philippines Manila, Manila, Philippines; ^2^Faculty of Education, Institute of Cognitive Neuroscience, Huaibei Normal University, Huaibei, China; ^3^Department of Psychological Medicine, Yong Loo Lin School of Medicine, National University of Singapore, Singapore, Singapore; ^4^Southeast Asia One Health University Network, Chiang Mai, Thailand; ^5^Department of Psychological Medicine, National University Health System, Singapore, Singapore; ^6^Institute of Health Innovation and Technology (iHealthtech), National University of Singapore, Singapore, Singapore

**Keywords:** anxiety, China, COVID-19, depression, middle-income, knowledge, precaution, Philippines

## Abstract

**Objective:** The differences between the physical and mental health of people living in a lower-middle-income country (LMIC) and upper-middle-income country (UMIC) during the COVID-19 pandemic was unknown. This study aimed to compare the levels of psychological impact and mental health between people from the Philippines (LMIC) and China (UMIC) and correlate mental health parameters with variables relating to physical symptoms and knowledge about COVID-19.

**Methods:** The survey collected information on demographic data, physical symptoms, contact history, and knowledge about COVID-19. The psychological impact was assessed using the Impact of Event Scale-Revised (IES-R), and mental health status was assessed by the Depression, Anxiety, and Stress Scale (DASS-21).

**Findings:** The study population included 849 participants from 71 cities in the Philippines and 861 participants from 159 cities in China. Filipino (LMIC) respondents reported significantly higher levels of depression, anxiety, and stress than Chinese (UMIC) during the COVID-19 (*p* < 0.01) while only Chinese respondents' IES-R scores were above the cut-off for PTSD symptoms. Filipino respondents were more likely to report physical symptoms resembling COVID-19 infection (*p* < 0.05), recent use of but with lower confidence on medical services (*p* < 0.01), recent direct and indirect contact with COVID (*p* < 0.01), concerns about family members contracting COVID-19 (*p* < 0.001), dissatisfaction with health information (*p* < 0.001). In contrast, Chinese respondents requested more health information about COVID-19. For the Philippines, student status, low confidence in doctors, dissatisfaction with health information, long daily duration spent on health information, worries about family members contracting COVID-19, ostracization, and unnecessary worries about COVID-19 were associated with adverse mental health. Physical symptoms and poor self-rated health were associated with adverse mental health in both countries (*p* < 0.05).

**Conclusion:** The findings of this study suggest the need for widely available COVID-19 testing in MIC to alleviate the adverse mental health in people who present with symptoms. A health education and literacy campaign is required in the Philippines to enhance the satisfaction of health information.

## Introduction

The World Health Organization (WHO) declared coronavirus disease 2019 (COVID-19) to be a Public Health Emergency of International Concern on January 30 (1) and a pandemic on March 11, 2020 (2). COVID-19 predominantly presents with respiratory symptoms (cough, sneezing, and sore throat), along with fever, fatigue and myalgia. It is thought to spread through droplets, contaminated surfaces, and asymptomatic individuals (3). By the end of April, over 3 million people have been infected globally (4).

The first country to identify the novel virus as the cause of the pandemic was China. The authorities responded with unprecedented restrictions on movement. The response included stopping public transport before Chinese New Year, an annual event that sees workers' mass emigration to their hometowns, and a lockdown of whole cities and regions (1). Two new hospitals specifically designed for COVID-19 patients were rapidly built in Wuhan. Such measures help slow the transmission of COVID-19 in China. As of May 2, there are 83,959 confirmed cases and 4,637 deaths from the virus in China (4). The Philippines was also affected early by the current crisis. The first case was suspected on January 22, and the country reported the first death from COVID-19 outside of mainland China (5). Similar to China, the Philippines implemented lockdowns in Manila. Other measures included the closure of schools and allowing arrests for non-compliance with measures (6). At the beginning of May, the Philippines recorded 8,772 cases and 579 deaths (4).

China was one of the more severely affected countries in Asia in the early stage of pandemic (7) while the Philippines is still experiencing an upward trend in the COVID-19 cases (6). The gross national income (GNI) per capita of the Philippines and China are USD 3,830 and 9,460, respectively, were classified with lower (LMIC) and upper-middle-income countries (UMIC) by the Worldbank (8). During the COVID-19 pandemic, five high-income countries (HIC), including the United States, Italy, the United Kingdom, Spain, and France, account for 70% of global deaths (9). The HIC faced the following challenges: (1) the lack of personal protection equipment (PPE) for healthcare workers; (2) the delay in response strategy; (3) an overstretched healthcare system with the shortage of hospital beds, and (4) a large number of death cases from nursing homes (10). The COVID-19 crisis threatens to hit lower and middle-income countries due to lockdown excessively and economic recession (11). A systematic review on mental health in LMIC in Asia and Africa found that LMIC: (1) do not have enough mental health professionals; (2) the negative economic impact led to an exacerbation of mental issues; (3) there was a scarcity of COVID-19 related mental health research in Asian LMIC (12). This systematic review could not compare participants from different middle-income countries because each study used different questionnaires. During the previous Severe Acute Respiratory Syndrome (SARS) epidemic, the promotion of protective personal health practices to reduce transmission of the SARS virus was found to reduce the anxiety levels in the community (13).

Before COVID-19, previous studies found that stress might be a modifiable risk factor for depression in LMICs (14) and UMICs (15–17). Another study involving thirty countries found that unmodifiable risk factors for depression included female gender, and depression became more common in 2004 to 2014 compared to previous periods (18). Further, there were cultural differences in terms of patient-doctor relationship and attitudes toward healthcare systems before the COVID-19 pandemic. In China, <20% of the general public and medical professionals view the doctor and patient relationship as harmonious (19). In contrast, Filipino seemed to have more trust and be compliant to doctors' recommendations (20). Patient satisfaction was more important than hospital quality improvement to maintain patient loyalty to the Chinese healthcare system (21). For Filipinos, improvement in the quality of healthcare service was found to improve patients' satisfaction (22).

Based on the above studies, we have the following research questions: (1) whether COVID-19 pandemic could be an important stressor and risk factor for depression for the people living in LMIC and UMIC (23), (2) Are physical symptoms that resemble COVID-19 infection and other concerns be risk factors for adverse mental health? (3) Are knowledge of COVID-19 and health information protective factors for mental health? (4) Would there be any cultural differences in attitudes toward doctors and healthcare systems during the pandemic between China and the Philippines? We hypothesized that UMIC (China) would have better physical and mental health than LMIC (the Philippines). The aims of this study were (a) to compare the physical and mental health between citizens from an LMIC (the Philippines) and UMIC (China); (b) to correlate psychological impact, depression, anxiety, and stress scores with variables relating to physical symptoms, knowledge, and concerns about COVID-19 in people living in the Philippines (LMIC) and China (UMIC).

## Methods

### Study Design and Study Population

We conducted a cross-cultural and quantitative study to compare Filipinos' physical and mental health with Chinese during the COVID-19 pandemic. The study was conducted from February 28 to March 1 in China and March 28 to April 7, 2020 in the Philippines, when the number of COVID-19 daily reported cases increased in both countries. The Chinese participants were recruited from 159 cities and 27 provinces. The Filipino participants, on the other hand, were recruited from 71 cities and 40 provinces representing the Luzon, Visayas, and Mindanao archipelago. A respondent-driven recruitment strategy was utilized in both countries. The recruitment started with a set of initial respondents who were associated with the Huaibei Normal University of China and the University of the Philippines Manila; who referred other participants by email and social network; these in turn refer other participants across different cities in China and the Philippines.

### Procedure

As both Chinese and Filipino governments recommended that the public minimize face-to-face interaction and isolate themselves during the study period, new respondents were electronically invited by existing study respondents. The respondents completed the questionnaires through an online survey platform (“SurveyStar,” Changsha Ranxing Science and Technology in China and Survey Monkey Online Survey in the Philippines). The Institutional Review Board of the University of Philippines Manila Research Ethics Board (UPMREB 2020-198-01) and Huaibei Normal University (China) approved the research proposal (HBU-IRB-2020-002). All respondents provided informed or implied consent. The collected data were anonymous and treated as confidential.

### Outcomes

This study used the National University of Singapore COVID-19 questionnaire, and its psychometric properties had been established in the initial phase of the COVID-19 epidemic (24). The National University of Singapore COVID-19 questionnaire consisted of questions that covered several areas: (1) demographic data; (2) physical symptoms related to COVID-19 in the past 14 days; (3) contact history with COVID-19 in the past 14 days; and (4) knowledge and concerns about COVID-19.

Demographic data about age, gender, education, household size, marital status, parental status, and residential city in the past 14 days were collected. Physical symptoms related to COVID-19 included breathing difficulty, chills, coryza, cough, dizziness, fever, headache, myalgia, sore throat, nausea, vomiting, and diarrhea. Respondents also rated their physical health status and stated their history of chronic medical illness. In the past 14 days, health service utilization variables included consultation with a doctor in the clinic, being quarantined by the health authority, recent testing for COVID-19 and medical insurance coverage. Knowledge and concerns related to COVID-19 included knowledge about the routes of transmission, level of confidence in diagnosis, source, and level of satisfaction of health information about COVID-19, the likelihood of contracting and surviving COVID-19 and the number of hours spent on viewing information about COVID-19 per day.

The psychological impact of COVID-19 was measured using the Impact of Event Scale-Revised (IES-R). The IES-R is a self-administered questionnaire that has been well-validated in the European and Asian population for determining the extent of psychological impact after exposure to a traumatic event (i.e., the COVID-19 pandemic) within one week of exposure (25, 26). This 22-item questionnaire, composed of three subscales, aims to measure the mean avoidance, intrusion, and hyperarousal (27). The total IES-R score is divided into 0–23 (normal), 24–32 (mild psychological impact), 33–36 (moderate psychological impact) and >37 (severe psychological impact) (28). The total IES-R score > 24 suggests the presence of post-traumatic stress disorder (PTSD) symptoms (29).

The respondents' mental health status was measured using the Depression, Anxiety, and Stress Scale (DASS-21) and the calculation of scores was based on a previous Asian study (30). DASS has been demonstrated to be a reliable and valid measure in assessing mental health in Filipinos (31–33) and Chinese (34, 35). IES-R and DASS-21 were previously used in research related to the COVID-19 epidemic (26, 36–38).

### Statistical Analysis

Descriptive statistics were calculated for demographic characteristics, physical symptom, and health service utilization variables, contact history variables, knowledge and concern variables, precautionary measure variables, and additional health information variables. To analyze the differences in the levels of psychological impact, levels of depression, anxiety and stress, the independent sample *t*-test was used to compare the mean score between the Filipino (LMIC) and Chinese (UMIC) respondents. The chi-squared test was used to analyze the differences in categorical variables between the two samples. We used linear regressions to calculate the univariate associations between independent and dependent variables, including the IES-S score and DASS stress, anxiety, and depression subscale scores for the Filipino and Chinese respondents separately with adjustment for age, marital status, and education levels. All tests were two-tailed, with a significance level of *p* < 0.05. Statistical analysis was performed on SPSS Statistic 21.0.

### Findings

#### Demographic Characteristics and Their Association With Psychological Impact and Adverse Mental Health Status

We received 849 responses from the Philippines and 861 responses from China for 1,710 individual respondents from both countries. The majority of Filipino respondents were women (71.0%), age between 22 and 30 years (26.6%), having a household size of 3–5 people (53.4%), high educational attainment (91.4% with a bachelor or higher degree), and married (68.9%). Similarly, the majority of Chinese respondents were women (75%), having a household size of 3–5 people (80.4%) and high educational attainment (91.4% with a bachelor or higher degree). There was a significantly higher proportion of Chinese respondents who had children younger than 16 years (*p* < 0.001) and student status (*p* < 0.001; See Table 1).

**Table 1:** Comparison of demographic characteristics between Filipino (LMIC) and Chinese (UMIC) respondents (*N* = 1,710).

**Demographics**	**The Philippines (LMIC) (*N* = 849)**	**China (UMIC) (*N* = 861)**	**Chi-square (χ^2^)**	***p*-value**
	***N*(%)**	***N*(%)**		
**Gender**				
Male	246(29.0)	215(25.0)	3.481	*p* = 0.062
Female	603(71.0)	646(75.0)		
**Age**				
[12–21]	230(27.1)	358(41.6)	252.337	*p* < 0.001^***^
[22–30]	226(26.6)	400(46.5)		
[31–40]	178(21.0)	37(4.3)		
[41–49]	129(15.2)	51(5.9)		
≥50	86(10.1)	15(1.7)		
**Parental Status**				
With child(ren) 16 years or below	132(15.5)	151(17.5)	484.778	*p* < 0.001^***^
With child(ren) above 16 years	55(6.5)	440(51.1)		
With child(ren) above 16 years, with child(ren) below 16 years	54(6.4)	0(0)		
No children/not applicable	608(71.6)	270(31.4)		
**Household Size**				
6 people or more	276(32.5)	124(14.4)	155.953	*p* < 0.001^***^
3–5 people	453(53.4)	692(80.4)		
2 people	67(7.9)	41(4.7)		
1 person	53(6.2)	4(0.5)		
**Educational Attainment**				
Primary school or below	5(0.6)	61(7.1)	95.986	*p* < 0.001^***^
Secondary School	68(8.0)	46(5.3)		
Bachelor's degree	544(64.1)	636(73.9)		
Masters/PhD	232(27.3)	118(13.7)		
**Employment Status**				
Student	296(34.9)	541(62.8)	163.641	*p* < 0.001^***^
Unemployed	31(3.6)	51(5.9)		
Housewife	26(3.1)	26(3.0)		
Retired	10(1.2)	3(0.4)		
Employed	486(57.2)	240(27.9)		
**Marital Status**				
Single	585(68.9)	134(15.6)	NA	NA
Married	240(28.3)	719(83.5)		
Divorced/Separated	19(2.2)	5(0.6)		
Widowed	5(0.6)	3(0.3)		

*** *p < 0.001, NA, due to too small number in one category and cannot perform Chi-square analysis*.

For Filipino respondents, the male gender and having a child were protective factors significantly associated with the lower score of IES-R (*p* < 0.05) and depression (*p* < 0.001), respectively. Single status was significantly associated with depression (*p* < 0.05), and student status was associated with higher IES-R, stress and depression scores (*p* < 0.01) (see Table 2). For Chinese respondents, the male gender was significantly associated with a lower score of IES-R but higher DASS depression scores (*p* < 0.01). Notwithstanding, there were other differences between Filipino and China respondents. Chinese respondents who stayed in a household with 3–5 people (*p* < 0.05) and more than 6 people (*p* < 0.05) were significantly associated with a higher score of IES-R as compared to respondents who stayed alone.

**Table 2:** Comparison of the association between demographic variables and the psychological impact as well as adverse mental health status between Filipino (LMIC) and Chinese (UMIC) respondents (*n* = 1,710).

**Demographics**	**The Philippines (LMIC)**								**China (UMIC)**							
	**Impact of event**		**Stress**		**Anxiety**		**Depression**		**Impact of event**		**Stress**		**Anxiety**		**Depression**	
	***B***	***t***	***B***	***t***	***B***	***t***	***B***	***T***	***B***	***t***	***B***	***t***	***B***	***t***	***B***	***t***
**Gender**																
Male	−0.17	−2.14^*^	−0.09	−1.40	−0.11	−1.10	−0.08	−0.81	−0.26	−2.61^**^	0.08	1.38	0.18	1.90	0.22	2.89^**^
Female	Reference		Reference		Reference		Reference		Reference		Reference		Reference		Reference	
**Age range (years)**																
(12–21)	0.39	2.91^**^	0.28	2.60^*^	0.48	2.96^**^	1.09	7.34^***^	0.77	2.28^*^	−0.03	−0.16	0.29	0.92	−0.02	−0.07
(22–30)	0.33	2.48^*^	0.28	2.59^*^	0.45	2.80^**^	0.67	4.54^***^	0.59	1.75	0.02	0.08	0.36	1.17	0.09	0.36
(31–40)	0.21	1.52	0.20	1.81	0.38	2.27^*^	0.18	1.16	0.63	1.62	−0.03	−0.15	0.29	0.80	0.03	0.12
(41–49)	−0.07	−0.50	−0.11	−0.94	0.04	0.20	−0.02	−0.12	0.26	0.70	−0.15	−0.70	−0.02	−0.05	−0.18	−0.63
>50	Reference		Reference		Reference		Reference		Reference		Reference		Reference		Reference	
**Marital Status**																
Single	−0.07	−0.15	0.08	0.21	0.23	0.39	1.08	1.97^*^	1.06	1.41	0.41	0.94	0.60	0.87	0.48	0.84
Married	−0.25	−0.52	−0.08	−0.19	−0.03	−0.05	0.49	0.89	1.27	1.71	0.46	1.06	0.80	1.17	0.58	1.02
Divorced or separated	−0.12	−0.22	−0.18	−0.41	0.10	0.15	0.53	0.86	1.27	1.35	0.60	1.10	1.00	1.16	0.60	0.84
Widowed	Reference		Reference		Reference		Reference		Reference		Reference		Reference		Reference	
**Parental Status**																
With child(ren) 16 years or below	−0.13	−1.30	−0.13	−1.63	−0.22	−1.78	−0.51	−4.39^***^	0.12	0.94	0.03	0.34	0.11	0.87	0.09	0.88
With child(ren) older than 16 years	−0.13	−0.84	−0.21	−1.73	−0.45	−2.50^*^	−0.65	−3.78^***^	0.10	0.96	0.003	0.06	−0.03	−0.34	−0.02	−0.29
No children/not applicable	Reference		Reference		Reference		Reference		Reference		Reference		Reference		Reference	
**Household size**																
6 people or more	−0.13	−0.84	0.19	1.46	−0.09	−0.49	0.04	0.23	1.44	2.20^*^	0.50	1.32	0.84	1.40	0.12	0.24
3–5 people	−0.15	−1.00	0.10	0.78	−0.16	−0.86	0.11	0.58	1.32	2.04^*^	0.45	1.19	0.77	1.29	0.06	0.13
2 persons	−0.10	−0.49	0.09	0.55	−0.09	−0.57	0.13	0.56	1.19	1.76	0.44	1.12	0.61	0.99	−0.16	−0.31
1 person	Reference		Reference		Reference		Reference		Reference		Reference		Reference		Reference	
**Employment status**																
Student	0.21	2.69^**^	0.19	3.01^**^	0.17	1.81	0.83	9.55^***^	−0.63	−0.85	−0.12	−0.27	−0.75	−1.10	−0.19	−0.34
Unemployed	−0.22	−1.11	0.02	0.12	−0.03	−0.11	0.18	0.80	0.06	0.30	0.10	0.91	0.29	1.67	0.32	2.25^*^
Housewife	0.24	1.13	0.05	0.31	−0.05	−0.20	−0.02	−0.09	−0.52	−1.99^*^	−0.03	−0.17	0.10	0.40	0.10	0.47
Retired	0.10	0.28	−0.06	−0.22	−0.40	−0.97	−0.30	−0.79	−0.16	−1.64	−0.01	−0.20	−0.01	−0.10	0.06	0.74
Employed	Reference		Reference		Reference		Reference		Reference		Reference		Reference		Reference	
**Education level**																
Secondary school	0.51	1.04	0.19	0.47	−0.39	−0.65	0.41	0.73	−0.06	−0.22	0.19	1.29	0.25	1.07	0.16	0.81
university/college	0.56	1.18	0.28	0.72	−0.38	−0.66	−0.004	−0.01	0.33	1.93	0.06	0.62	0.04	0.26	−0.01	−0.09
Masters/PhD	0.29	0.60	0.13	0.34	−0.65	−1.12	−0.51	−0.92	0.24	1.18	0.13	1.11	0.24	1.31	0.14	0.89
Primary school or below	Reference		Reference		Reference		Reference		Reference		Reference		Reference		Reference	

* *p < 0.05*;

** *p < 0.01*;

*** *p < 0.001*.

#### Comparison Between the Filipino (LMIC) and Chinese (UMIC) Respondents and Their Mental Health Status

**Figure 1:**
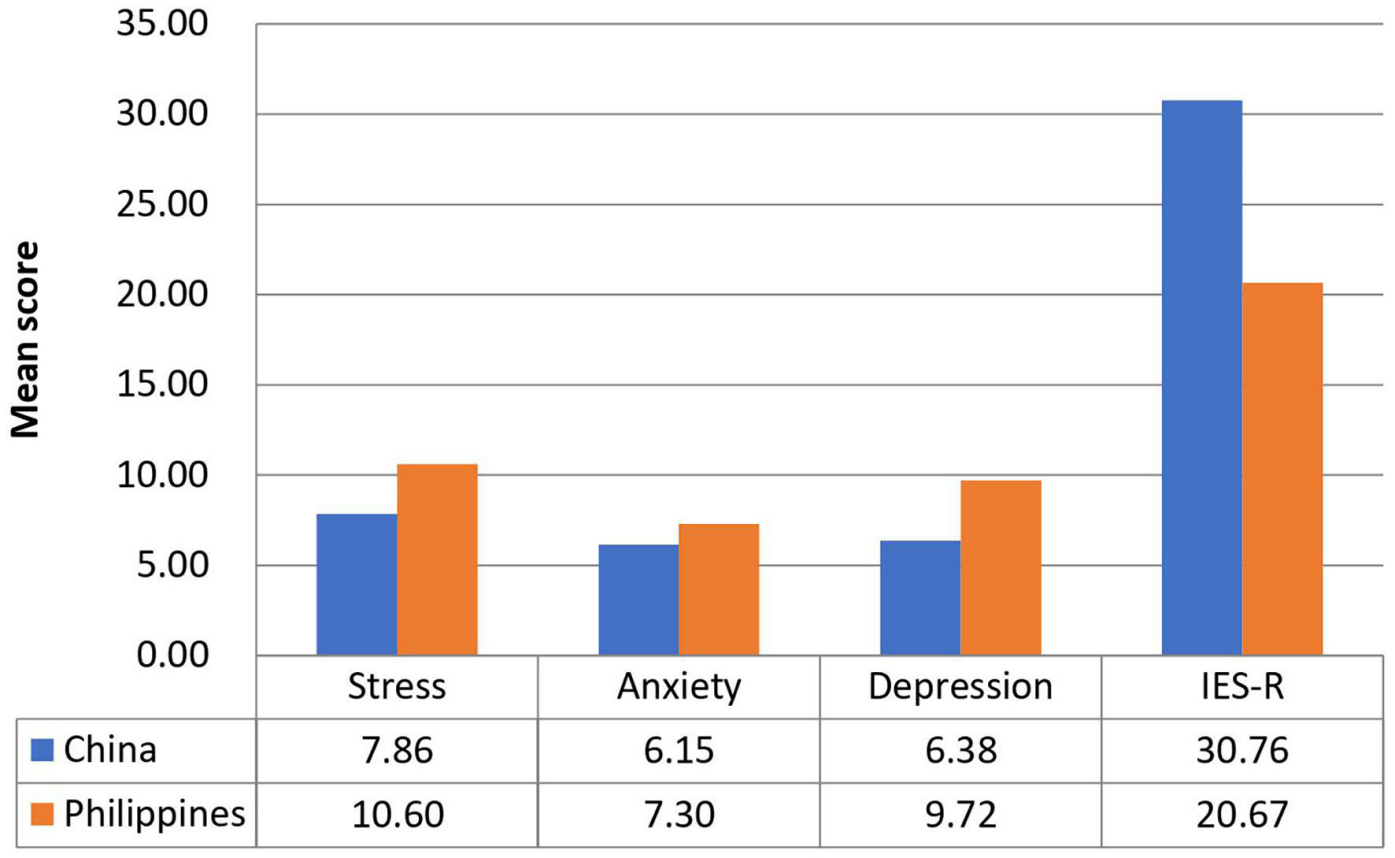
Comparison of the mean scores of DASS-stress, anxiety and depression subscales, and IES-R scores between Filipino and Chinese respondents.

Figure 1 compares the mean scores of DASS-stress, anxiety, and depression subscales and IES-R scores between the Filipino and Chinese respondents. For the DASS-stress subscale, Filipino respondents reported significantly higher stress (*p* < 0.001), anxiety (*p* < 0.01), and depression (*p* < 0.01) than Chinese (UMIC). For IES-R, Filipino (LMIC) had significantly lower scores than Chinese (*p* < 0.001). The mean IES-R scores of Chinese were higher than 24 points, indicating the presence of PTSD symptoms in Chinese respondents only.

#### Physical Symptoms, Health Status, and Its Association With Psychological Impact and Adverse Mental Health Status

There were significant differences between Filipino (LMIC) and Chinese (UMIC) respondents regarding physical symptoms resembling COVID-19 and health status. There was a significantly higher proportion of Filipino respondents who reported headache (*p* < 0.001), myalgia (*p* < 0.001), cough (*p* < 0.001), breathing difficulty (*p* < 0.001), dizziness (*p* < 0.05), coryza (*p* < 0.001), sore throat (*p* < 0.001), nausea and vomiting (*p* < 0.001), recent consultation with a doctor (*p* < 0.01), recent hospitalization (*p* < 0.001), chronic illness (*p* < 0.001), direct (*p* < 0.001), and indirect (*p* < 0.001) contact with a confirmed diagnosis of COVID-19 as compared to Chinese (see Supplementary Table 1). Significantly more Chinese respondents were under quarantine (*p* < 0.001).

Linear regression showed that headache, myalgia, cough, dizziness, coryza as well as poor self-rated physical health were significantly associated with higher IES-R scores, DASS-21 stress, anxiety, and depression subscale scores in both countries after adjustment for confounding factors (*p* < 0.05; see Table 3). Furthermore, breathing difficulty, sore throat, and gastrointestinal symptoms were significantly associated with higher DASS-21 stress, anxiety and depression subscale scores in both countries (*p* < 0.05). Chills were significantly associated with higher DASS-21 stress and depression scores (*p* < 0.01) in both countries. Recent quarantine was associated with higher DASS-21 subscale scores in Chinese respondents only (*p* < 0.05).

**Table 3:** Association between physical health status and contact history and the perceived impact of COVID-19 outbreak as well as adverse mental health status during the epidemic after adjustment for age, gender, and marital status (*n* = 1,710).

**Symptoms**	**The Philippines (LMIC) (*****n*** **=** **849)**								**China (UMIC) (*****n*** **=** **861)**							
	**Impact of Event**		**Stress**		**Anxiety**		**Depression**		**Impact of Event**		**Stress**		**Anxiety**		**Depression**	
	**B**	**t**	**B**	**t**	**B**	**t**	**B**	**t**	**B**	**t**	**B**	**T**	**B**	**t**	**B**	**t**
**Persistent/recurrent fever**																
Yes	0.33	0.88	0.11	0.35	0.27	0.60	−0.04	−0.10	1.49	1.16	3.52	4.74^***^	3.11	2.64^**^	3.32	3.43^**^
No	Reference		Reference		Reference		Reference		Reference		Reference		Reference		Reference	
**Chills**																
Yes	0.40	1.68	0.61	3.16^**^	0.52	1.79	0.76	2.88^**^	0.60	1.61	0.90	4.17^***^	0.84	2.44^*^	0.90	3.18^**^
No	Reference		Reference		Reference		Reference		Reference		Reference		Reference		Reference	
**Headache**																
Yes	0.37	4.43^***^	0.38	5.64^***^	0.63	6.34^***^	0.35	3.76^***^	0.57	2.92^**^	0.48	4.21^***^	0.80	4.42^***^	0.55	3.71^***^
No	Reference		Reference		Reference		Reference		Reference		Reference		Reference		Reference	
**Myalgia**																
Yes	0.54	4.91^***^	0.52	5.82^***^	0.82	6.17^***^	0.40	3.19^**^	0.48	2.75^**^	0.41	4.06^***^	0.58	3.59^***^	0.58	4.37^***^
No	Reference		Reference		Reference		Reference		Reference		Reference		Reference		Reference	
**Cough**																
Yes	0.37	3.66^***^	0.22	2.68^**^	0.53	4.37^***^	0.13	1.13	0.54	2.17^*^	0.61	4.26^***^	0.67	2.95^**^	0.66	3.55^***^
No	Reference		Reference		Reference		Reference		Reference		Reference		Reference		Reference	
**Breathing difficulty**																
Yes	0.67	4.84^***^	0.69	6.17^***^	1.27	7.71^***^	0.64	4.12^***^	0.78	1.49	1.04	3.39^**^	1.01	2.10^*^	1.40	3.54^***^
No	Reference		Reference		Reference		Reference		Reference		Reference		Reference		Reference	
**Dizziness**																
Yes	0.74	4.78^***^	0.64	5.06^***^	0.91	4.80^***^	0.67	3.83^***^	0.92	3.94^***^	0.82	6.07^***^	0.97	4.52^***^	0.69	3.91^***^
No	Reference		Reference		Reference		Reference		Reference		Reference		Reference		Reference	
**Coryza**																
Yes	0.20	1.88	0.18	2.05^*^	0.31	2.40^*^	0.26	2.13^*^	0.59	3.02^**^	0.34	2.94^**^	0.52	2.91^**^	0.56	3.77^***^
No	Reference		Reference		Reference		Reference		Reference		Reference		Reference		Reference	
**Sore throat**																
Yes	0.30	2.76^**^	0.43	4.85^***^	0.61	4.72^***^	0.26	2.16^*^	0.33	1.44	0.59	4.40^***^	0.77	3.63^***^	0.73	4.22^***^
No	Reference		Reference		Reference		Reference		Reference		Reference		Reference		Reference	
**Persistent fever and cough or breathing difficulty**																
Yes	−0.62	−0.59	−0.64	−0.74	1.08	0.85	−0.55	−0.47	1.49	1.16	3.52	4.74^***^	3.11	2.64^**^	3.32	3.43^**^
No	Reference		Reference		Reference		Reference		Reference		Reference		Reference		Reference	
**Nausea, vomiting, or diarrhea**																
Yes	0.83	3.91^***^	0.84	4.86^***^	1.32	5.14^***^	0.89	3.74^***^	0.74	1.51	1.26	4.47^***^	1.33	2.99^**^	1.26	3.42^**^
No	Reference		Reference		Reference		Reference		Reference		Reference		Reference		Reference	
**Consultation with a doctor**																
Yes	0.05	0.28	0.06	0.42	0.38	1.74	0.11	0.52	0.47	1.29	0.22	1.06	0.32	0.95	0.22	0.80
No	Reference		Reference		Reference		Reference		Reference		Reference		Reference		Reference	
**Tested for COVID**–**19**																
Yes	−0.66	−1.54	0.07	0.20	0.25	0.48	−0.31	−0.65	−0.40	−0.62	−0.20	−0.54	−0.03	−0.06	−0.06	−0.12
No	Reference		Reference		Reference		Reference		Reference		Reference		Reference		Reference	
**Recent quarantine**																
Yes	0.04	0.14	0.23	1.02	0.45	1.35	0.17	0.55	0.35	1.74	0.25	2.07^*^	0.53	2.84^**^	0.37	2.42^*^
No	Reference		Reference		Reference		Reference		Reference		Reference		Reference		Reference	
**Self**–**evaluation of health**																
Poor	1.47	3.54^***^	1.83	5.41^***^	1.89	3.73^***^	2.02	4.34^***^	0.78	2.15^*^	1.09	5.20^***^	1.51	4.58^***^	1.21	4.49^***^
Fair	0.70	7.18^***^	0.55	6.93^***^	0.83	7.08^***^	0.76	7.02^***^	0.40	4.28^***^	0.23	4.31^***^	0.44	5.15^***^	0.40	5.77^***^
Good	Reference		Reference		Reference		Reference		Reference		Reference		Reference		Reference	
**Medical insurance**																
Yes	0.02	0.29	0.04	0.59	−0.09	−0.97	0.01	0.08	−0.03	−0.16	−0.18	−2.01^*^	−0.37	−2.61^**^	−0.22	−1.88
No	Reference		Reference		Reference		Reference		Reference		Reference		Reference		Reference	
**Chronic illness**																
Yes	0.02	0.17	0.14	1.74	0.07	0.57	0.14	1.28	0.43	2.14^*^	0.24	2.02^*^	0.24	1.32	0.27	1.79
No	Reference		Reference		Reference		Reference		Reference		Reference		Reference		Reference	
**Direct contact with a confirmed case of COVID-19**																
Yes	−0.06	−0.25	0.17	0.86	0.04	0.13	0.26	0.98	−1.54	−1.69	0.16	0.29	0.47	0.56	0.64	0.93
No	Reference		Reference		Reference		Reference		Reference		Reference		Reference		Reference	
**Indirect contact with a confirmed case of COVID-19**																
Yes	−0.03	−0.18	0.11	0.81	0.02	0.11	−0.16	−0.84	−0.33	−0.63	0.10	0.31	0.19	0.39	0.23	0.58
No	Reference		Reference		Reference		Reference		Reference		Reference		Reference		Reference	
**Contact with (COVID-19) contaminated materials**																
Yes	0.34	1.05	0.58	2.20^*^	0.34	0.86	0.26	0.72	−1.10	−1.72	0.11	0.29	0.36	0.61	0.31	0.63
No	Reference		Reference		Reference		Reference		Reference		Reference		Reference		Reference	

* *p < 0.05*;

** p < 0.01; and

*** *p < 0.001*.

#### Perception, Knowledge, and Concerns About COVID-19 and Its Association With Psychological Impact and Adverse Mental Health Status

Filipino (LMIC) and Chinese (UMIC) respondents held significantly different perceptions in terms of knowledge and concerns related to COVID-19 (see Supplementary Table 2). For the routes of transmission, there were significantly more Filipino respondents who agreed that droplets transmitted the COVID-19 (*p* < 0.001) and contact via contaminated objects (*p* < 0.001), but significantly more Chinese agreed with the airborne transmission (*p* < 0.001). For the detection and risk of contracting COVID-19, there were significantly more Filipino who were not confident about their doctor's ability to diagnose COVID-19 (*p* < 0.001). There were significantly more Filipino respondents who were worried about their family members contracting COVID-19 (*p* < 0.001). For health information, there were significantly more Filipino who were unsatisfied with the amount of health information (*p* < 0.001) and spent more than three hours per day on the news related to COVID-19 (*p* < 0.001). There were significantly more Chinese respondents who felt ostracized by other countries (*p* < 0.001).

Linear regression analysis after adjustment of confounding factors showed that the Filipino and Chinese respondents showed different findings (see Table 4). Chinese respondents who reported a very low perceived likelihood of contracting COVID-19 were significantly associated with lower DASS depression scores (*p* < 0.05). There were similarities between the two countries. Filipino and Chinese respondents who perceived a very high likelihood of survival were significantly associated with lower DASS-21 depression scores (*p* < 0.05). Regarding the level of confidence in the doctor's ability to diagnose COVID-19, both Filipino and Chinese respondents who were very confident in their doctors were significantly associated with lower DASS-21 depression scores (*p* < 0.01). Filipino and Chinese respondents who were satisfied with health information were significantly associated with lower DASS-21 anxiety and depression scores (*p* < 0.01). Chinese and Filipino respondents who were worried about their family members contracting COVID-19 were associated with higher IES-R and DASS-21 subscale scores (*p* < 0.05). In contrast, only Filipino respondents who spent <1 h per day monitoring COVID-19 information was significantly associated with lower IES-R and DASS-21 stress and anxiety scores (*p* < 0.05). Filipino respondents who felt ostracized were associated with higher IES-R and stress scores (*p* < 0.05).

**Table 4:** Comparison of association of knowledge and concerns related to COVID-19 with mental health status after adjustment for age, gender, and marital status (*N* = 1,710).

	**The Philippines (LMIC) (*****N*** **=** **849)**								**China (UMIC) (*****N*** **=** **861)**							
**Perception, knowledge and concerns related to COVID-19**	**Impact of event**		**Stress**		**Anxiety**		**Depression**		**Impact of event**		**Stress**		**Anxiety**		**Depression**	
	***B***	***t***	***B***	***t***	***B***	***t***	***B***	***t***	***B***	***t***	***B***	***t***	***B***	***t***	***B***	***t***
**Route of transmission Droplets**																
Agree	−0.69	−1.59	0.10	0.28	0.08	0.15	0.05	0.11	0.003	0.02	−0.12	−1.44	−0.15	−1.14	−0.16	−1.39
Disagree	−0.56	−0.82	−0.001	−0.002	1.14	1.38	0.45	0.60	0.06	0.14	0.45	1.89	0.67	1.78	0.51	1.65
Do not know	Reference		Reference		Reference		Reference		Reference		Reference		Reference		Reference	
**Contact via contaminated objects**																
Agree	−0.08	−0.30	−0.08	−0.38	0.15	0.46	−0.32	−1.10	−0.07	−0.64	−0.08	−1.21	−0.07	−0.63	−0.11	−1.34
Disagree	0.20	0.48	0.02	0.06	0.12	0.24	−0.34	−0.72	0.11	0.55	0.004	0.03	−0.18	−1.00	−0.15	−1.02
Do not know	Reference		Reference		Reference		Reference		Reference		Reference		Reference		Reference	
**Airborne**																
Agree	−0.08	−0.68	0.11	1.21	0.02	0.17	0.12	0.98	0.07	0.62	0.04	0.58	0.01	0.12	−0.04	−0.46
Disagree	−0.02	−0.15	0.10	1.09	0.03	0.24	0.20	1.70	−0.02	−0.13	−0.05	−0.61	−0.06	−0.51	−0.09	−0.83
Do not know	Reference		Reference		Reference		Reference		Reference		Reference		Reference		Reference	
**Level of confidence in a doctor's ability to diagnose or recognize COVID-19**																
Very confident	−0.35	−2.33^*^	−0.16	−1.27	−0.29	−1.58	−0.35	−2.11^*^	0.02	0.06	−0.29	−1.44	−0.39	−1.25	−0.81	−3.14^**^
Fairly confident	−0.25	−1.74	−0.03	−0.23	−0.01	−0.03	−0.29	−1.80	0.32	0.92	−0.21	−1.04	−0.30	−0.94	−0.68	−2.60^**^
Not very confident	−0.01	−0.05	0.15	1.09	0.16	0.80	0.16	0.87	0.39	0.89	0.04	0.17	0.07	0.17	−0.45	−1.36
Not confident	0.07	0.24	0.12	0.49	−0.03	−0.09	0.08	0.25	−0.15	−0.15	−0.19	−0.32	0.63	0.70	−0.22	−0.30
Do not know	Reference		Reference		Reference		Reference		Reference		Reference		Reference		Reference	
**Likelihood of contracting COVID−19 during the pandemic**																
Very likely	0.17	0.91	0.14	0.90	0.19	0.85	0.13	0.61	−0.24	−1.24	−0.08	−0.70	0.04	0.25	−0.11	−0.75
Fairly likely	0.09	0.60	0.17	1.42	0.14	0.76	0.18	1.05	−0.02	−0.16	−0.14	−1.61	−0.05	−0.32	−0.16	−1.35
Not very likely	−0.08	−0.55	−0.10	−0.85	−0.19	−1.06	0.001	0.01	0.03	0.22	−0.16	−1.86	−0.10	−0.76	−0.16	−1.48
Not likely	−0.05	−0.30	−0.11	−0.87	−0.10	−0.49	0.01	0.04	−0.13	−0.74	−0.19	−1.86	−0.15	−0.95	−0.31	−2.29^*^
Do not know	Reference		Reference		Reference		Reference		Reference		Reference		Reference		Reference	
**Likelihood of surviving if infected with COVID-19**																
Very likely	−0.10	−0.70	−0.06	−0.50	−0.11	−0.61	−0.32	−1.98^*^	−0.27	−1.92	−0.14	−1.73	−0.18	−1.41	−0.24	−2.27^*^
Fairly likely	−0.13	−0.89	−0.10	−0.82	−0.12	−0.69	−0.28	−1.73	−0.01	−0.12	−0.08	−1.15	−0.10	−0.91	−0.19	−1.99^*^
Not very likely	0.27	1.44	0.23	1.52	0.33	1.45	0.29	1.37	0.02	0.12	0.05	0.47	0.39	2.17^*^	0.20	1.33
Not likely	0.55	1.93	0.31	1.33	0.34	0.98	0.55	1.72	−0.23	−0.65	0.07	0.36	0.52	1.62	0.16	0.62
Do not know	Reference		Reference		Reference		Reference		Reference		Reference		Reference		Reference	
**Satisfaction with the amount of health information available about COVID-19**																
Very satisfied	−1.31	−3.46^**^	−0.50	−1.58	−1.33	−2.87^**^	−1.37	−3.24^**^	−0.07	−0.28	−0.60	−3.94^***^	−0.71	−2.95^**^	−0.73	−3.67^***^
Fairly satisfied	−1.18	−3.21^**^	−0.46	−1.50	−1.27	−2.83^**^	−1.18	−2.86^**^	0.31	1.21	−0.48	−3.21^**^	−0.62	−2.63^**^	−0.61	−3.11^**^
Not very satisfied	−0.94	−2.50^*^	−0.34	−1.10	−1.14	−2.49^*^	−0.93	−2.23^*^	0.11	0.34	−0.33	−1.81	−0.51	−1.74	−0.38	−1.57
Not satisfied	−0.62	−1.49	−0.06	−0.18	−0.48	−0.95	−0.29	−0.62	0.82	2.20^*^	0.06	0.25	0.13	0.37	−0.19	−0.69
Do not know	Reference		Reference		Reference		Reference		Reference		Reference		Reference		Reference	
**Degree of worry about family members being diagnosed with COVID-19**																
Very worried	0.60	2.13^*^	0.51	2.20^*^	0.66	1.95	0.77	2.43^*^	−0.004	−0.01	−0.53	−2.49^*^	−0.39	−1.15	−0.77	−2.79^**^
Fairly worried	0.21	0.73	0.26	1.12	0.12	0.34	0.47	1.46	0.08	0.22	−0.63	−2.97^**^	−0.39	−1.15	−0.79	−2.86^**^
Not very worried	0.05	0.15	0.32	1.15	0.25	0.61	0.47	1.24	−0.36	−0.97	−0.76	−3.50^***^	−0.66	−1.94	−0.94	−3.35^**^
Not worried	−0.17	−0.32	−0.17	−0.38	−0.45	−0.69	0.35	0.58	−0.43	−1.08	−0.70	−3.04^**^	−0.63	−1.73	−0.92	−3.04^**^
No other family members	Reference		Reference		Reference		Reference		Reference		Reference		Reference		Reference	
**Hours spent daily on the news relating to COVID-19**																
≦1	−0.42 to −4.43^***^		−0.23 to −2.91^**^		−0.34 to −2.91^**^		−0.23 to −2.13^*^		0.06 to 0.49		0.06 to 0.76		−0.03 to −0.28		−0.05 to −0.52	
(1–3)	−0.04 to −0.46		0.04 to 0.54		−0.17 to −1.65		0.10 to 1.05		−0.03 to −0.25		−0.06 to −0.97		−0.09 to −0.97		−0.06 to −0.78	
≦3	Reference		Reference		Reference		Reference		Reference		Reference		Reference		Reference	
**Feeling ostracized by other countries with the outbreak of COVID-19**																
Yes	0.38 4.04^***^		0.17 2.10^*^		0.16 1.38		0.15 1.44		0.16 1.69		0.10 1.80		0.05 0.63		0.08 1.18	
No	Reference		Reference		Reference		Reference		Reference		Reference		Reference		Reference	

* *p < 0.05*;

** *p < 0.01*;

*** *p < 0.001*.

#### Health Information About COVID-19 and Its Association With Psychological Impact and Adverse Mental Health Status

Filipino (LMIC) and Chinese (UMIC) respondents held significantly different views on the information required about COVID-19. There were significantly more Chinese respondents who needed information on the symptoms related to COVID-19, prevention methods, management and treatment methods, regular information updates, more personalized information, the effectiveness of drugs and vaccines, number of infected by geographical locations, travel advice and transmission methods as compared to Filipino (*p* < 0.01; See Supplementary Table 3). In contrast, there were significantly more Filipino respondents who needed information on other countries' strategies and responses than Chinese (*p* < 0.001).

Information on management methods and transmission methods were significantly associated with higher IES-R scores in Chinese respondents (*p* < 0.05; see Table 5). Travel advice, local transmission data, and other countries' responses were significantly associated with lower DASS-21 stress and depression scores in Chinese respondents only (*p* < 0.05). There was only one significant association observed in Filipino respondents; information on transmission methods was significantly associated with lower DASS-21 depression scores (*p* < 0.05).

**Table 5:** Comparison of the association between information needs about COVID-19 and the psychological impact as well as adverse mental health status between Filipino (LMIC) and Chinese (UMIC) participants after adjustment for age, gender, and marital status (*N* = 1,710).

**Health information required**	**The Philippines (LMIC) (*****N*****=** **849)**								**China (UMIC) (*****N*** **=** **861)**							
	**Impact of event**		**Stress**		**Anxiety**		**Depression**		**Impact of event**		**Stress**		**Anxiety**		**Depression**	
	***B***	***t***	***B***	***t***	***B***	***t***	***B***	***t***	***B***	***t***	***B***	***t***	***B***	***t***	***B***	***t***
**Symptoms related to COVID-19 infection**																
Yes	0.08	1.01	0.01	0.08	0.08	0.83	−0.10	−1.15	0.20	1.63	0.04	0.52	0.12	1.02	0.02	0.19
No	Reference		Reference		Reference		Reference		Reference		Reference		Reference		Reference	
**Prevention methods**																
Yes	0.01	0.08	−0.03	−0.52	−0.001	−0.01	−0.17	−1.92	0.25	1.71	−0.10	−1.14	−0.16	−1.14	−0.15	−1.33
No	Reference		Reference		Reference		Reference		Reference		Reference		Reference		Reference	
**Management/Treatment methods**																
Yes	0.09	1.11	0.04	0.53	0.04	0.42	−0.08	−0.87	0.24	2.30^*^	0.09	1.45	0.20	2.10^*^	0.09	1.11
No	Reference		Reference		Reference		Reference		Reference		Reference		Reference		Reference	
**Regular information update**																
Yes	0.04	0.50	0.02	0.33	0.03	0.31	−0.09	−0.97	0.39	1.89	−0.14	−1.15	−0.13	−0.67	−0.20	−1.26
No	Reference		Reference		Reference		Reference		Reference		Reference		Reference		Reference	
**Local transmission data**																
Yes	0.01	0.11	−0.02	−0.27	−0.02	−0.19	−0.12	−1.35	0.04	0.19	−0.34	−2.60^*^	−0.31	−1.50	−0.36	−2.12^*^
No	Reference		Reference		Reference		Reference		Reference		Reference		Reference		Reference	
**More personalized information, such as those with pre-existing medical conditions**																
Yes	0.09	1.18	0.03	0.46	0.07	0.74	−0.08	−0.90	0.03	0.19	−0.13	−1.63	−0.17	−1.32	−0.19	−1.83
No	Reference		Reference		Reference		Reference		Reference		Reference		Reference		Reference	
**Effectiveness of drugs and vaccines**																
Yes	0.08	0.94	0.02	0.33	0.04	0.36	−0.08	−0.84	0.10	0.61	−0.18	−1.80	−0.11	−0.69	−0.23	−1.81
No	Reference		Reference		Reference		Reference		Reference		Reference		Reference		Reference	
**Infection statistics by geographical location**																
Yes	0.02	0.27	0.01	0.16	0.05	0.47	−0.13	−1.41	0.14	0.82	−0.09	−0.92	−0.16	−1.05	−0.19	−1.49
No	Reference		Reference		Reference		Reference		Reference		Reference		Reference		Reference	
**Travel advice**																
Yes	0.01	0.13	−0.01	−0.09	0.02	0.22	−0.10	−1.24	0.11	0.83	−0.17	−2.09^*^	−0.07	−0.56	−0.25	−2.46^*^
No	Reference		Reference		Reference		Reference		Reference		Reference		Reference		Reference	
**Transmission methods**																
Yes	−0.004	−0.05	−0.04	−0.62	0.02	0.21	−0.19	−2.19^*^	0.48	2.66^**^	−0.11	−1.03	−0.17	−1.01	−0.14	−1.03
No	Reference		Reference		Reference		Reference		Reference		Reference		Reference		Reference	
**Strategies and responses from other countries**																
Yes	0.06	0.77	0.04	0.61	0.07	0.68	−0.08	−0.95	0.26	2.85^**^	−0.01	−0.22	−0.13	−1.47	−0.15	−2.07^*^
No	Reference		Reference		Reference		Reference		Reference		Reference		Reference		Reference	

* *p < 0.05*;

** *p < 0.01*.

## Discussion

To our best knowledge, this is the first study that compared the physical and mental health as well as knowledge, attitude and belief about COVID-19 between citizens from an LMIC (The Philippines) and UMIC (China). Filipino respondents reported significantly higher levels of depression, anxiety and stress than Chinese during the COVID-19, but only the mean IES-R scores of Chinese respondents were above the cut-off scores for PTSD symptoms. Filipino respondents were more likely to report physical symptoms resembling COVID-19 infection, recent use of medical services with lower confidence, recent direct, and indirect contact with COVID, concerns about family members contracting COVID-19 and dissatisfaction with health information. In contrast, Chinese respondents requested more health information about COVID-19 and were more likely to stay at home for more than 20–24 h per day. For the Filipino, student status, low confidence in doctors, unsatisfaction of health information, long hours spent on health information, worries about family members contracting COVID-19, ostracization, unnecessary worries about COVID-19 were associated with adverse mental health.

The most important implication of the present study is to understand the challenges faced by a sample of people from an LMIC (The Philippines) compared to a sample of people from a UMIC (China) in Asia. As physical symptoms resembling COVID-19 infection (e.g., headache, myalgia, dizziness, and coryza) were associated with adverse mental health in both countries, this association could be due to lack of confidence in healthcare system and lack of testing for coronavirus. Previous research demonstrated that adverse mental health such as depression could affect the immune system and lead to physical symptoms such as malaise and other somatic symptoms (39, 40). Based on our findings, the strategic approach to safeguard physical and mental health for middle-income countries would be cost-effective and widely available testing for people present with COVID-19 symptoms, providing a high quality of health information about COVID-19 by health authorities.

Students were afraid that confinement and learning online would hinder their progress in their studies (41). This may explain why students from the Philippines reported higher levels of IES-R and depression scores. Schools and colleges should evaluate the blended implementation of online and face-to-face learning to optimize educational outcomes when local spread is under control. As a significantly higher proportion of Filipino respondents lack confidence in their doctors, health authorities should ensure adequate training and develop hospital facilities to isolate COVID-19 cases and prevent COVID-19 spread among healthcare workers and patients (42). Besides, our study found that Filipino respondents were dissatisfied with health information. In contrast, Chinese respondents demanded more health information related to COVID-19. The difference could be due to stronger public health campaign launched by the Chinese government including national health education campaigns, a health QR (Quick Response) code system and community engagement that effectively curtailed the spread of COVID-19 (43). The high expectation for health information could be explained by high education attainment of participants as about 91.4 and 87.6% of participants from China and the Philippines have a university education.

Furthermore, the governments must employ communication experts to craft information, education, and messaging materials that are target-appropriate to each level of understanding in the community. That the Chinese Government rapidly deployed medical personnel and treated COVID-19 patients at rapidly-built hospitals (44) is in itself a confidence-building measure. Nevertheless, recent quarantine was associated with higher DASS-21 subscale scores in Chinese respondents only. It could be due to stricter control and monitoring of movements imposed by the Chinese government during the lockdown (45). Chinese respondents who stayed with more than three family members were associated with higher IES-R scores. The high IES-R scores could be due to worries of the spread of COVID-19 to family members and overcrowded home environment during the lockdown. The Philippines also converted sports arena into quarantine/isolation areas for COVID-19 patients with mild symptoms. These prompt actions helped restore public confidence in the healthcare system (46). A recent study reported that cultural factors, demand pressure for information, the ease of information dissemination via social networks, marketing incentives, and the poor legal regulation of online contents are the main reasons for misinformation dissemination during the COVID-19 pandemic (47). Bastani and Bahrami (47) recommended the engagement of health professionals and authorities on social media during the pandemic and the improvement of public health literacy to counteract misinformation.

Chinese respondents were more likely to feel ostracized and Filipino respondents associated ostracization with adverse mental health. Recently, the editor-in-chief of *The Lancet*, Richard Horton, expressed concern of discrimination of a country or particular ethnic group, saying that while it is important to understand the origin and inter-species transmission of the coronavirus, it was both unhelpful and unscientific to point to a country as the origin of the Covid-19 pandemic, as such accusation could be highly stigmatizing and discriminatory (48). The global co-operation involves an exchange of expertise, adopting effective prevention strategies, sharing resources, and technologies among UMIC and LIMC to form a united front on tackling the COVID-19 pandemic remains a work in progress.

### Strengths and Limitations

The main strength of this study lay in the fact that we performed in-depth analysis and studied the relationship between physical and mental outcomes and other variables related to COVID-19 in the Philippines and China. However, there are several limitations to be considered when interpreting the results. Although the Philippines is a LMIC and China is a UMIC, the findings cannot be generalized to other LIMCs and UMICs. Another limitation was the potential risk of sampling bias. This bias could be due to the online administration of questionnaires, and the majority of respondents from both countries were respondents with good educational attainment and internet access. We could not reach out to potential respondents without internet access (e.g., those who stayed in the countryside or remote areas). Further, our findings may not be generalizable to other middle-income countries.

## Conclusion

During the COVID-19 pandemic, Filipinos (LMIC) respondents reported significantly higher levels of depression, anxiety and stress than Chinese (UMIC). Filipino respondents were more likely to report physical symptoms resembling COVID-19 infection, recent use of medical services with lower confidence, recent direct and indirect contact with COVID, concerns about family members contracting COVID-19 and dissatisfaction with health information than Chinese. For the current COVID-19 and future pandemic, Middle income countries need to adopt the strategic approach to safeguard physical and mental health by establishing cost-effective and widely available testing for people who present with COVID-19 symptoms; provision of high quality and accurate health information about COVID-19 by health authorities. Our findings urge middle income countries to prevent ostracization of a particular ethnic group, learn from each other, and unite to address the challenge of the COVID-19 pandemic and safeguard physical and mental health.

## Data Availability Statement

The raw data supporting the conclusions of this article will be made available by the authors, without undue reservation.

## Ethics Statement

Ethical review and approval was required for the study on human participants in accordance with the local legislation and institutional requirements. Written informed consent to participate in this study was provided by the participants' legal guardian/next of kin. The Institutional Review Board of the University of Philippines Manila Research Ethics Board (UPMREB 2020- 198-01) and Huaibei Normal University (China) approved the research proposal (HBU-IRB-2020-002).

## Author Contributions

Concept and design: CW, MT, CT, RP, VK, and RH. Acquisition, analysis, and interpretation of data: CW, MT, CT, RP, LX, CHa, XW, YT, and VK. Drafting of the manuscript: CW, MT, CT, RH, and JA. Critical revision of the manuscript: MT, CT, CHo, and JA. Statistical analysis: CW, PR, RP, LX, XW, and YT. All authors contributed to the article and approved the submitted version.

## Conflict of Interest

The authors declare that the research was conducted in the absence of any commercial or financial relationships that could be construed as a potential conflict of interest.
