# Supplementary material for: Impact of the COVID-19 Pandemic on Physical and Mental Health in Lower and Upper Middle-Income Asian Countries: A Comparison Between the Philippines and China
Source: Front Psychiatry. 2021 Feb 9;11:568929. doi: 10.3389/fpsyt.2020.568929 (PMC7901572; doi:10.3389/fpsyt.2020.568929)
Supplement: Supplementary file 1 [file Table_1.DOCX]

| **Supplementary Table 1. Comparison of physical symptoms related to COVID-19, health status and contact history between Filipino (LMIC) and Chinese (UMIC) respondents (n =1710) .** | | | | | |
| --- | --- | --- | --- | --- | --- |
| **Physical Symptoms and Health Status** | **The Philippines (LMIC)**（N=849） |  | **China**  **(UMIC)**  (N=861) | Chi-square (χ^2^) | *p*-value |
|  | N(%) |  | N(%) |  |  |
| **Persistent/Recurrent Fever** |  |  |  |  |  |
| Yes | 8(0.9) |  | 1(0.1) | NA | NA |
| No | 841(99.1) |  | 860(99.9) |  |  |
| **Chills** |  |  |  |  |  |
| Yes | 20(2.4) |  | 12(1.4) | 2.154 | p=0.142 |
| No | 829(97.6) |  | 849(98.6) |  |  |
| **Headache** |  |  |  |  |  |
| Yes | 212(25.0) |  | 45(5.2) | 130.491 | p<0.001*** |
| No | 637(75.0) |  | 816(94.8) |  |  |
| **Myalgia** |  |  |  |  |  |
| Yes | 101(11.9) |  | 57(6.6) | 14.190 | p<0.001*** |
| No | 748(88.1) |  | 804(93.4) |  |  |
| **Cough** |  |  |  |  |  |
| Yes | 128(15.1) |  | 28(3.3) | 72.094 | p<0.001*** |
| No | 721(84.9) |  | 833(96.7) |  |  |
| **Breathing difficulties** |  |  |  |  |  |
| Yes | 61(7.2) |  | 6(0.7) | 47.800 | p<0.001*** |
| No | 788(92.8) |  | 855(99.3) |  |  |
| **Dizziness** |  |  |  |  |  |
| Yes | 48(5.7) |  | 31(3.6) | 4.090 | p=0.043* |
| No | 801(94.3) |  | 830(96.4) |  |  |
| **Coryza** |  |  |  |  |  |
| Yes | 112(13.2) |  | 46(5.3) | 31.407 | p<0.001*** |
| No | 737(86.8) |  | 815(94.7) |  |  |
| **Sore Throat** |  |  |  |  |  |
| Yes | 108(12.7) |  | 32(3.7) | 46.108 | p<0.001*** |
| No | 741(87.3) |  | 829(96.3) |  |  |
| **Persistent fever with cough or breathing difficulties** |  |  |  |  |  |
| Yes | 1(0.1) |  | 1(0.1) | NA | NA |
| No | 848(99.9) |  | 860(99.9) |  |  |
| **Nausea, vomiting or diarrhea** |  |  |  |  |  |
| Yes | 25(2.9) |  | 7（0.8） | 10.578 | p=0.001** |
| No | 824(97.1) |  | 854（99.2） |  |  |
| **Doctor consultation in an outpatient clinic in the last 14 days** |  |  |  |  |  |
| Yes | 35(4.1) |  | 13(1.5) | 10.695 | p=0.001** |
| No | 814(95.9) |  | 848(98.5) |  |  |
| **Hospitalized in the last 14 days** |  |  |  |  |  |
| Yes | 4(0.5) |  | 861(100) | 1694.073 | p<0.001*** |
| No | 845(99.5) |  | 0(0) |  |  |
| **Tested for COVID-19 in the last 14 days** |  |  |  |  |  |
| Yes | 6(0.7) |  | 4(0.5) | NA | NA |
| No | 843(99.3) |  | 857(99.5) |  |  |
| **Quarantine in the last 14 days** |  |  |  |  |  |
| Yes | 15(1.8) |  | 42(4.9) | 12.842 | p<0.001*** |
| No | 834(98.2) |  | 819(95.1) |  |  |
| **Chronic illness** |  |  |  |  |  |
| Yes | 151(17.8) |  | 43(5.0) | 69.542 | p<0.001*** |
| No | 698(82.2) |  | 818(95.0) |  |  |
| **Medical Insurance** |  |  |  |  |  |
| Yes | 468(55.1) |  | 785(91.2) | 283.684 | p<0.001*** |
| No | 381(44.9) |  | 76(8.8) |  |  |
| **Direct contact with patients with a confirmed diagnosis of COVID-19** |  |  |  |  |  |
| Yes | 21(2.5) |  | 2(0.2) | 16.182 | p<0.001*** |
| No | 828(97.5) |  | 859(99.8) |  |  |
| **Indirect contact with patients with a confirmed diagnosis of COVID-19** |  |  |  |  |  |
| Yes | 41(4.8) |  | 6(0.7) | 27.309 | p<0.001*** |
| No | 808(95.2) |  | 855(99.3) |  |  |
| **Contact with materials contaminated by COVID-19** |  |  |  |  |  |
| Yes | 11(1.3) |  | 4(0.5) | 3.396 | p=0.065 |
| No | 838(98.7) |  | 857(99.5) |  |  |

*p<0.05, **p<0.01, ***p<0.001

**Supplementary Table 2. Comparison of knowledge and concerns related to COVID-19 and the psychological impact as well as adverse mental health status between Filipino (LMIC) and Chinese (UMIC) respondents (N=1710).**

| Knowledge and concerns related to COVID-19 | | **The Philippines (LMIC)**（N=849） |  | | **China (UMIC)**  (N=861) | Chi-square (χ^2^) | p-value |
| --- | --- | --- | --- | --- | --- | --- | --- |
|  |  | N(%) |  | | N(%) |  |  |
| **Route of transmission** | | | | | | | |
| **Droplets** | | | | | | | |
| Agree | 839(98.8) | |  | 762(88.5) | | 78.422 | p<0.001*** |
| Disagree | 4(0.5) | |  | 11(1.3) | |  |  |
| Do not know | 6(0.7) | |  | 88(10.2) | |  |  |
| **Contact via**  **contaminated objects** | | | | | | | |
| Agree | 822(96.8) | |  | 634（73.6） | | 182.351 | p<0.001*** |
| Disagree | 10(1.2) | |  | 57（6.6） | |  |  |
| Do not know | 17(2.0) | |  | 170（19.8） | |  |  |
| **Airborne** | | | | | | | |
| Agree | 289(34.1) | |  | 498（57.8） | | 184.959 | p<0.001*** |
| Disagree | 429(50.5) | |  | 166（19.3） | |  |  |
| Do not know | 131(15.4) | |  | 197（22.9） | |  |  |
| **Level of confidence in doctor’s ability to**  **diagnose or recognize COVID-19** | | | | | | | |
| Very confident | 231(27.2) | |  | 498（57.9） | | 217.580 | p<0.001*** |
| Somewhat confident | 422(49.7) | |  | 325（37.8） | |  |  |
| Not very confident | 118(13.9) | |  | 21（2.4） | |  |  |
| Not confident | 16(1.9) | |  | 2（0.2） | |  |  |
| Do not know | 62(7.3) | |  | 15（1.7） | |  |  |
| **Likelihood of contracting COVID−19 during the pandemic** | | | | | | | |
| Very likely | 66(7.8) | |  | 78（9.1） | | 18.346 | P=0.001** |
| Somewhat likely | 270(31.8) | |  | 231（26.8） | |  |  |
| Not very likely | 315(37.1) | |  | 336（39.0） | |  |  |
| Not likely | 137(16.1) | |  | 112（13.0） | |  |  |
| Do not know | 61(7.2) | |  | 104（12.1） | |  |  |
| **Likelihood of surviving if infected with COVID-19** | | | | | | | |
| Very likely | 320(37.7) | |  | 197（22.9） | | 65.702 | p<0.001*** |
| Somewhat likely | 387(45.6) | |  | 445（51.7） | |  |  |
| Not very likely | 64(7.5) | |  | 62（7.2） | |  |  |
| Not likely | 17(2.0) | |  | 15（1.7） | |  |  |
| Do not know | 61(7.2) | |  | 142（16.5） | |  |  |
| **Satisfaction with the amount of**  **health information available about COVID-19** | | | | | | | |
| Very satisfied | 125(14.7) | |  | 247（28.7） | | 122.608 | p<0.001*** |
| Somewhat satisfied | 518(61.0) | |  | 522（60.6） | |  |  |
| Not very satisfied | 169(19.9) | |  | 45（5.2） | |  |  |
| Not satisfied | 29(3.4) | |  | 21（2.5） | |  |  |
| Do not know | 8(1.0) | |  | 26（3.0） | |  |  |
| **Degree of worry about family members**  **being diagnosed with COVID-19** | | | | | | | |
| Very worried | 512(60.3) | |  | 282（32.8） | | 223.539 | p<0.001*** |
| Fairly worried | 289(34.0) | |  | 327（38.0） | |  |  |
| Not very worried | 29(3.4) | |  | 181（21.0） | |  |  |
| Not worried | 5(0.6) | |  | 58（6.7） | |  |  |
| No other family members | 14(1.7) | |  | 13（1.5） | |  |  |
| **Feeling ostracized by other countries**  **with the outbreak of COVID-19** | | | | | | | |
| Yes | 146(17.2) | |  | 300(34.8) | | 69.047 | p<0.001*** |
| No | 703(82.8) | |  | 561(65.2) | |  |  |
| **Hours spent daily on news relating to COVID-19** | | | | | | | |
| [0-1] | 178(21.0) | |  | 564(65.5) | | 477.019 | p<0.001*** |
| (1-3] | 270(31.8) | |  | 248(28.8) | |  |  |
| >3 | 401(47.2) | |  | 49(5.7) | |  |  |

*p<0.05, **p<0.01, ***p<0.001

| **Supplementary Table 3. Comparison of information needs about COVID-19 between Filipino (LMIC) and Chinese (UMIC) respondents (N = 1710).** | | | | | | |
| --- | --- | --- | --- | --- | --- | --- |
| Health information needed | | **The Philippines**（N=849） |  | **China**  (N=861) | Chi-square (χ^2^) | p-value |
|  |  | N(%) |  | N(%) |  |  |
| **Symptoms** | | | | | | |
| Yes | 581(68.4) | |  | 736(85.5) | 70.195 | p<0.001*** |
| No | 268(31.6) | |  | 125(14.5) |  |  |
| **Prevention methods** | | | | | | |
| Yes | 582(68.6) | |  | 778(90.4) | 124.900 | p<0.001*** |
| No | 267(31.4) | |  | 83(9.6) |  |  |
| **Management/Treatment methods** | | | | | | |
| No | 606(71.4) | |  | 663(77.0) | 7.068 | p=0.008** |
| No | 243(28.6) | |  | 198(23.0) |  |  |
| **Regular information update** | | | | | | |
| Yes | 601(70.8) | |  | 820(95.2) | 181.943 | p<0.001*** |
| No | 248(29.2) | |  | 41(4.8) |  |  |
| **Local transmission data** | | | | | | |
| Yes | 590(69.5) | |  | 826(95.9) | 209.926 | p<0.001*** |
| No | 259(30.5) | |  | 35(4.1) |  |  |
| **More personalized information,**  **such as for those with pre-existing medical conditions** | | | | | | |
| Yes | 577(68.0) | |  | 765(88.9) | 110.432 | p<0.001*** |
| No | 272(32.0) | |  | 96(11.1) |  |  |
| **Effectiveness of drugs and vaccines** | | | | | | |
| Yes | 611(72.0) | |  | 798(92.7) | 126.485 | p<0.001*** |
| No | 238(28.0) | |  | 63(7.3) |  |  |
| **Infection statistics by geographical location** | | | | | | |
| Yes | 586(69.0) | |  | 798(92.7) | 155.097 | p<0.001*** |
| No | 263(31.0) | |  | 63(7.3) |  |  |
| **Travel advice** | | | | | | |
| Yes | 517(60.9) | |  | 760(88.3) | 169.400 | p<0.001*** |
| No | 332(39.1) | |  | 101(11.7) |  |  |
| **Transmission methods** | | | | | | |
| Yes | 566(66.7) | |  | 807(93.7) | 197.839 | p<0.001*** |
| No | 283(33.3) | |  | 54(6.3) |  |  |
| **Strategies and responses by other countries** | | | | | | |
| Yes | 587(69.1) | |  | 305(35.4) | 194.745 | p<0.001*** |
| No | 262(30.9) | |  | 556(64.6) |  |  |

***p<0.001
